# Internal-salt-regulated stability of brine-in-oil emulsions: interfacial mechanisms and bulk-property effects

**DOI:** 10.1039/d6ra05935j

**Published:** 2026-09-07

**Authors:** Panhong Yu, Yang Xue, Chao Li, Sunan Wang, Huaike Li, Tie Geng, Hong Sui, Lin He

**Affiliations:** a Oilfield Chemical Division, China Oilfield Services Limited Yanjiao Hebei 065201 China; b School of Chemical Engineering and Technology, Tianjin University Tianjin 300354 China linhe@tju.edu.cn

## Abstract

Water-in-oil (W/O) emulsions are widely encountered in petroleum production, separation processes, and functional emulsion formulations. In practical systems, the dispersed aqueous phase commonly contains dissolved salts, whereas their influence on W/O emulsion stability is still not fully understood. This review therefore summarizes the effects of internal salts on W/O emulsion stability from a mechanism-oriented perspective. Internal salts can interact with interfacially active species and thereby regulate their interfacial adsorption and organization, which is reflected in changes in interfacial tension and interfacial viscoelasticity. They can also modify electrostatic interactions between droplets, altering attractive or repulsive forces during droplet approach and coalescence. In addition, salt-induced changes in density, emulsion viscosity, and solubility can affect gravitational sedimentation, droplet migration, and collision kinetics, while limited solubility may cause crystallization and disturb emulsion homogeneity. Finally, current challenges and future perspectives are discussed, including coupled characterization, realistic-condition testing, and predictive formulation design. This review provides a framework for linking salt composition, interfacial mechanisms, bulk-property effects, and W/O emulsion stability.

## Abstract 2

Internal salts regulate W/O emulsion stability by modulating interfacial adsorption, film properties, and droplet coalescence. Their effects depend on salt concentration, ion identity, and composition, together with associated bulk-phase properties.

## Graphical abstract

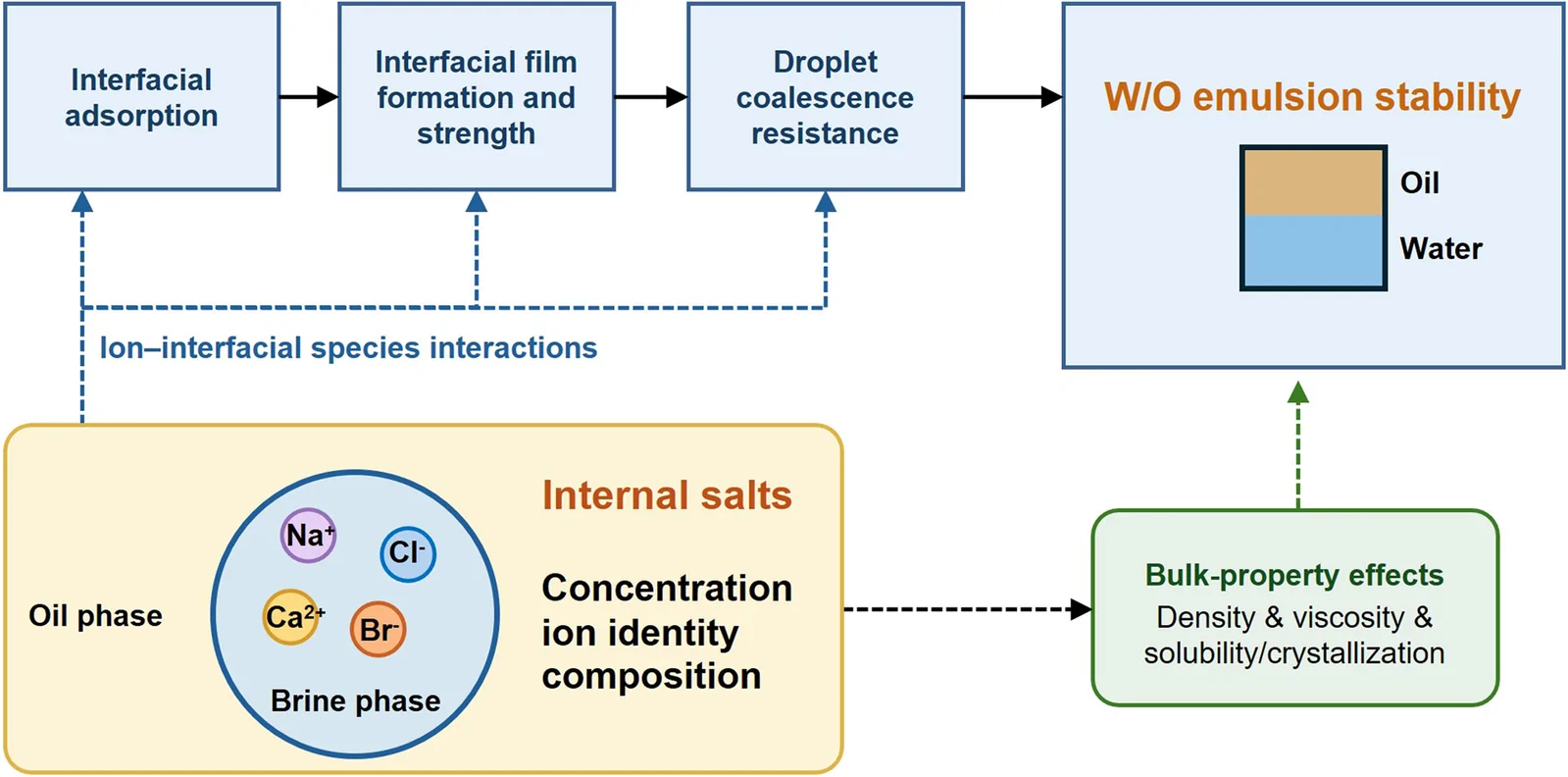

## 1 Introduction

Water-in-oil (W/O) emulsions are widely encountered in petroleum and other industrial systems.^1–3^ They are formed when aqueous droplets are dispersed in a continuous oil phase under mechanical shear, turbulence, or mixing, and are stabilized by interfacially active species adsorbed at the oil–water interface.^4,5^ These stabilizers/emulsifiers, including surfactants, polymers, and particles, can reduce interfacial tension and form protective interfacial films that hinder droplet coalescence.^6,7^ Depending on the application, W/O emulsion stability can be either desirable or problematic. It is required in formulated systems such as drilling and completion fluids and enhanced oil recovery, where stability maintains phase homogeneity and fluid performance.^8–11^ In contrast, unintentionally formed W/O emulsions during crude oil production, transportation, and refining can hinder oil–water separation and create challenges in dehydration, desalting, and wastewater treatment.^12–15^

In practical W/O emulsions, the dispersed aqueous phase is rarely pure water. Instead, it commonly contains dissolved inorganic salts.^16^ These salts may originate from formation water, produced water, injected seawater, drilling and completion brines, or saline industrial effluents.^17,18^ In petroleum-related systems, common internal salts include NaCl, KCl, CaCl_2_, MgCl_2_, CaBr_2_, and mixed brines.^19^ Their concentrations can vary widely with reservoir conditions, processing history, and formulation requirements.^20^ These salts determine the ionic strength, water activity, osmotic pressure, density, and viscosity of the internal phase.^2,21^ They can also alter the interfacial environment experienced by emulsifiers and droplets.^22,23^ Therefore, internal salts are not merely background components of the dispersed phase. They are important formulation and environmental variables that regulate W/O emulsion stability.

However, the influence of internal salts on W/O emulsion stability remains insufficiently understood.^24,25^ Salt effects are not governed by salinity or ion valence alone. They are also coupled with ion identity, emulsifier type, oil composition, temperature, interfacial film properties, and aqueous-phase properties.^26,27^ As a result, salts may enhance emulsion stability by promoting interfacial adsorption or strengthening interfacial films.^28,29^ They may also induce destabilization by causing emulsifier aggregation, film disruption, or unfavorable changes in droplet interactions.^30,31^ Many studies mainly evaluate macroscopic stability indicators, such as phase separation, droplet size, or electrical stability.^32,33^ In contrast, the underlying interfacial mechanisms are still not clearly established.^34^ These mechanisms include emulsifier adsorption, interfacial film mechanics, and droplet coalescence behavior.^35–37^ Therefore, a mechanism-oriented understanding of how internal salts regulate W/O emulsion stability is needed.

**Fig. 1:**
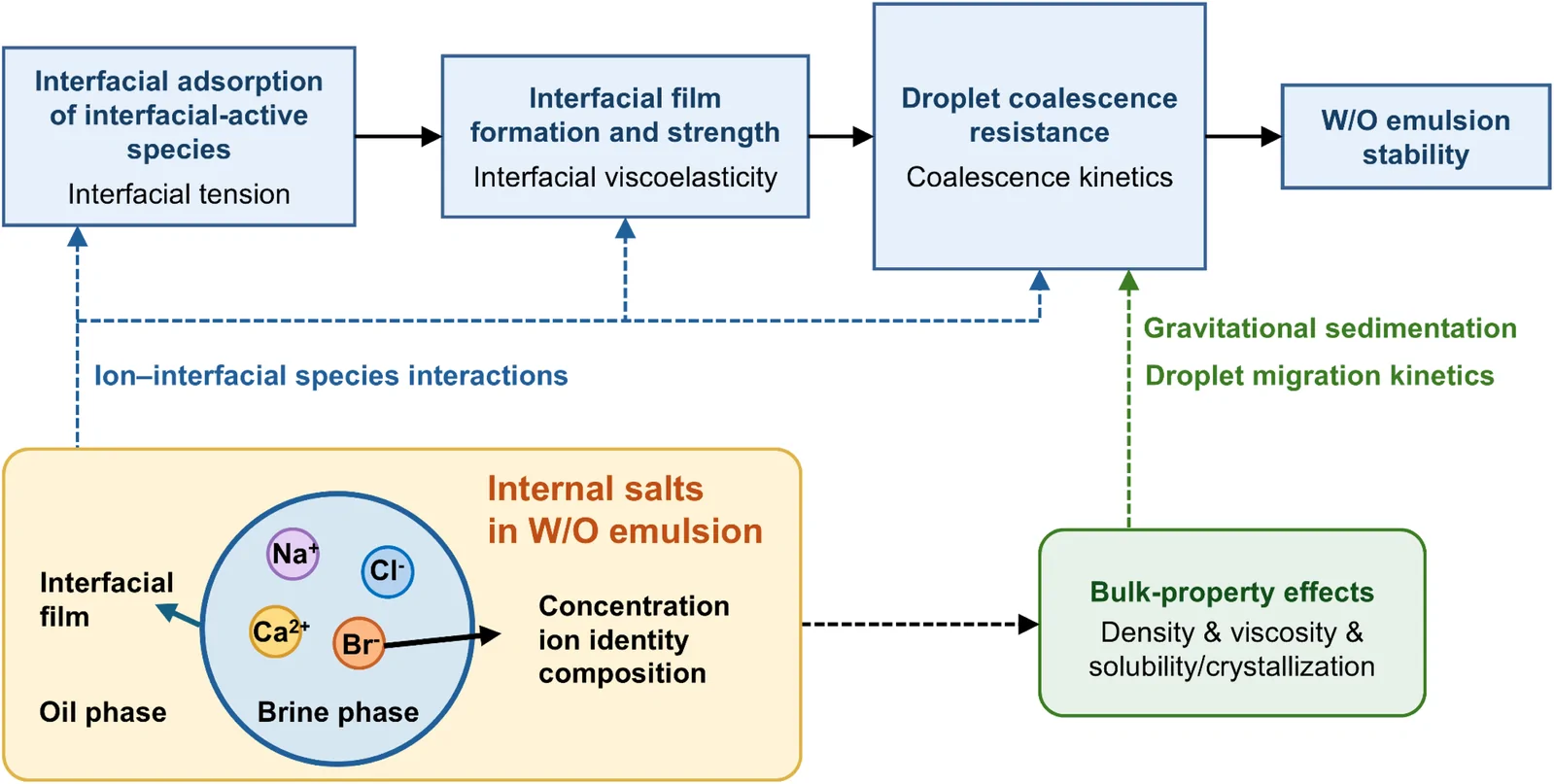
Mechanistic framework of internal-salt-regulated W/O emulsion stability.

Against this background, the effects of internal salts on W/O emulsion stability are reviewed from a mechanism-oriented perspective. As shown in Fig. 1, salt regulation is discussed across the stabilization process, from interfacial film formation and resistance to droplet coalescence to macroscopic emulsion stability. The potential interplay among different regulatory pathways is further considered to identify current mechanistic gaps and challenges. Overall, this review provides an integrated mechanistic perspective for understanding and controlling salt-regulated W/O emulsions, with potential relevance to other salt-containing W/O formulations.

## 2 Effects of internal salts on interfacial adsorption and interfacial tension

Interfacial adsorption is one of the earliest processes through which internal salts regulate W/O emulsion formation.^38^ Interfacially active species, such as surfactants, asphaltenes, resins, polymers, or particles, adsorb at the oil–water interface to reduce interfacial free energy and form interfacial films.^39^ Changes in oil–water interfacial tension therefore provide a useful macroscopic indication of salt-regulated adsorption behavior. In this section, internal-salt effects on interfacial adsorption are discussed from three aspects: salt concentration, particle- or solid-mediated interfacial organization, and ion-specific interactions.

**Fig. 2:**
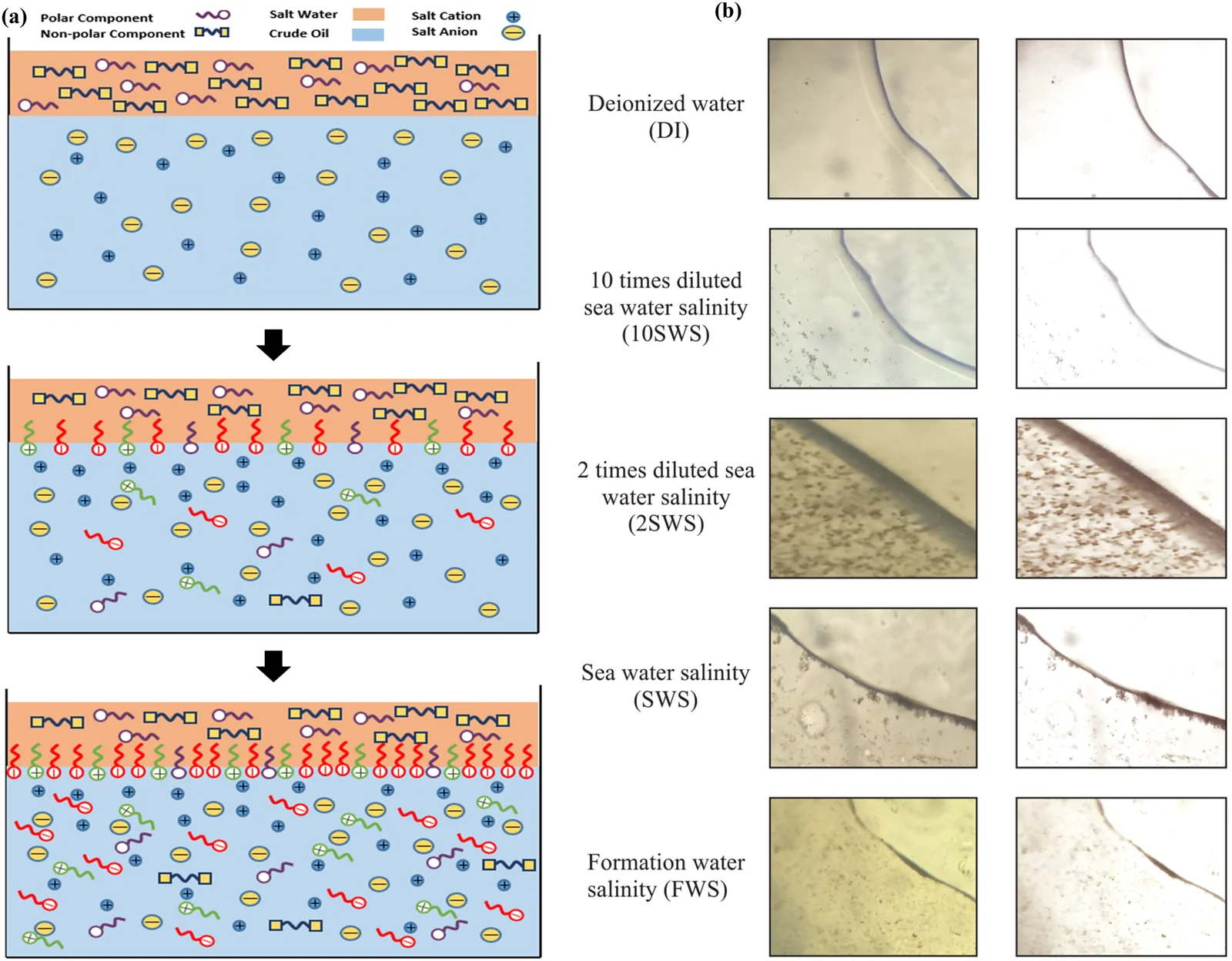
Regulation of oil–water interfacial tension by internal salts. (a) The mechanism of the interaction between ions and surfactant components at the oil–water interface. Reproduced with permission from ref. 40. Copyright 2022, Wiley. (b) Microscopy images at the interfaces between synthetic oil and different brine: salt effect on the adsorption of interfacially active species. Reproduced with permission from ref. 41. Copyright 2019, Elsevier.

Salt concentration is a primary factor governing the oil–water interfacial tension in W/O emulsion systems. At low to moderate ionic strength (typically on the order of 10^−2^ to 10^−1^ M), dissolved ions can screen electrostatic repulsion among charged or polar interfacial species (Fig. 2a), thereby facilitating the adsorption of interfacially active species at the oil–water interface (Fig. 2b). This enhanced interfacial accumulation usually lowers the interfacial tension.^42^

**Fig. 3:**
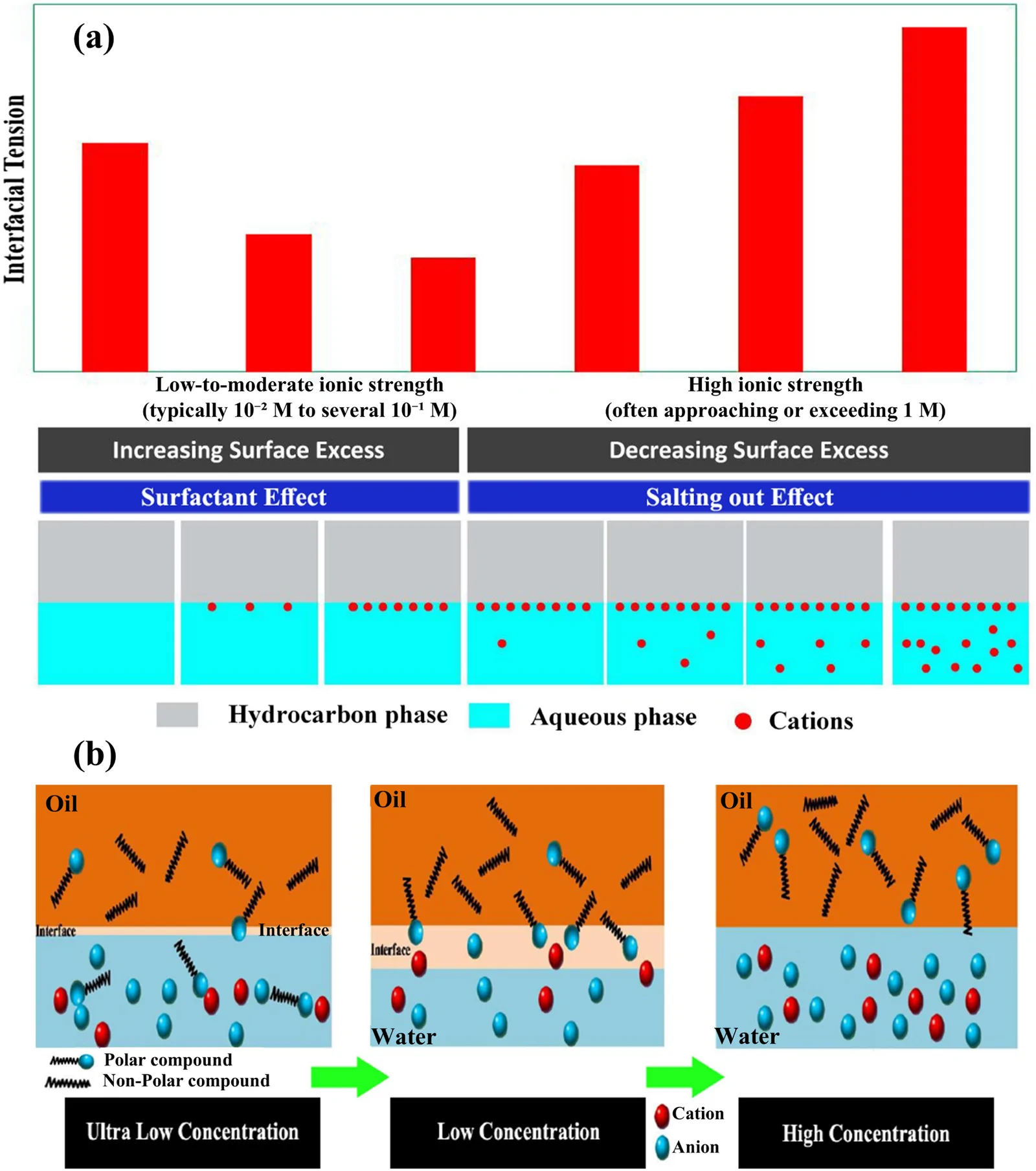
Schematic mechanisms of salinity-dependent interfacial tension behavior at the oil–water interface. (a) Nonmonotonic variation of interfacial tension with brine concentration. (b) Schematic representation of the oil–brine interface at different salinity levels, showing salinity-dependent redistribution and organization of polar interfacial species. Reproduced with permission from ref. 45. Copyright 2020, Elsevier.

However, the effect of salinity is rarely linear. After an optimum salinity is reached, further salt addition may lead to an IFT plateau or even an increase because of interfacial saturation, interfacial rearrangement, reduced emulsifier solubility, or bulk aggregation.^43,44^ This nonmonotonic behavior has been explained by the competition between salt-induced interfacial accumulation and salt-induced depletion of surface-active species. As schematically illustrated in Fig. 3a, when salt is first introduced into the aqueous phase, cations can promote the migration and adsorption of polar crude-oil components, especially asphaltenes or resin-like species, toward the oil–water interface. This “salting-in”-like effect increases the excess surface concentration of interfacially active species and leads to a decrease in interfacial tension. Near an optimum salinity, the interfacial accumulation reaches a maximum and the interfacial tension reaches a minimum. Further increasing salt concentration may reverse this trend. At high salinity, with ionic strength approaching or exceeding 1 M, electrostatic screening, cation saturation, salting-out effects, or bulk aggregation can reduce the effective interfacial concentration of surface-active species, resulting in an increase in interfacial tension. A complementary schematic description is shown in Fig. 3b. With increasing brine concentration, the distribution of polar oil components between the aqueous phase, oil phase, and interface changes. Under low-salinity conditions (typically with ionic strength ranging from 10^−2^ M to several 10^−1^ M), polar components are more likely to accumulate at, or remain close to, the interface, whereas high salinity suppresses their dispersion into the aqueous phase and changes their interfacial arrangement. Therefore, the minimum salinity-dependent interfacial tension should be understood as a result of competing interfacial adsorption and depletion processes, rather than as a simple linear salt effect.

**Fig. 4:**
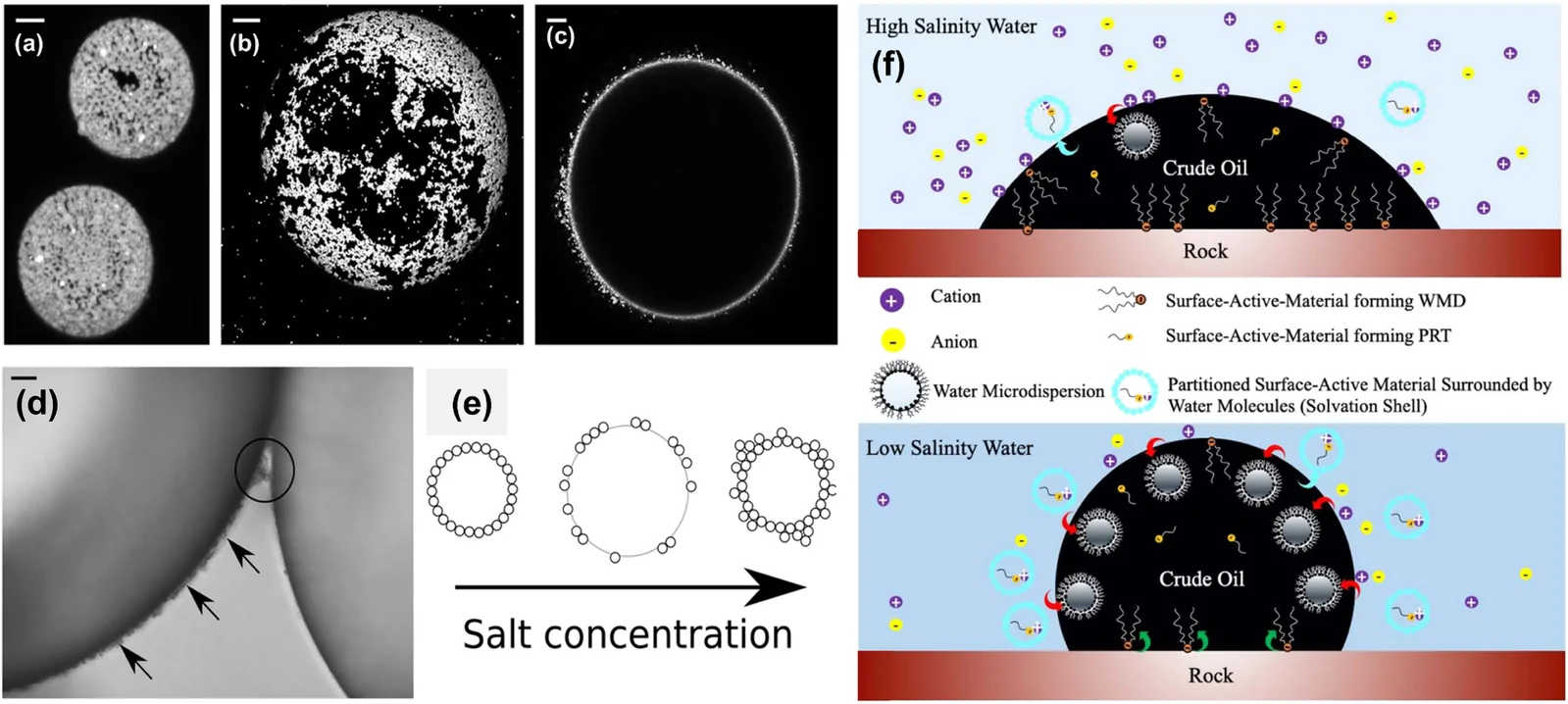
Salt-regulated interfacial adsorption and organization in particle-containing and oil–water–solid systems. (a–e) Effects of NaCl concentration on particle coverage and organization at droplet interfaces. Reproduced with permission from ref. 46. Copyright 2020, Royal Society of Chemistry. (a) At 10 mM NaCl, particles form a relatively compact monolayer at the oil–water interface. (b) At 30 mM NaCl, partial surface coverage and particle aggregation appear at the droplet interface. (c) At 100 mM NaCl, the interfacial particle layer becomes thickened. (d) At 300 mM NaCl, the aggregated particle layer protrudes into the continuous phase and can connect neighboring droplets. (e) The cartoon summarizes the transition from monolayer coverage to partial coverage and finally to a thickened interfacial particle network with increasing salinity. (f) Schematic illustration of salinity-dependent redistribution of surface-active materials in an oil–water–solid system. High-salinity and low-salinity conditions can change the partitioning, microdispersion, and competitive adsorption of surface-active species between the oil–water interface and solid surfaces. Reproduced with permission from ref. 47. Copyright 2021, American Chemical Society.

Salt-regulated interfacial adsorption becomes more complex when solid particles or mineral surfaces are present. In this case, surface-active species may be redistributed among the oil–water interface, solid surfaces, and bulk phases, while particles can also adsorb at droplet interfaces and form interfacial layers. Fig. 4a–e shows an O/W Pickering emulsion containing nanoparticles, with NaCl dissolved in the continuous aqueous phase. The surface coverage and organization of particles at droplet interfaces changed markedly with NaCl concentration. At very low NaCl concentration, droplets were almost fully covered by a particle monolayer. At intermediate salinity, partial surface coverage and particle aggregation were observed. At higher salinity, the particle layer extended into the aqueous phase, indicating a thickened and less efficient stabilizing layer. This behavior suggests that salt-induced charge screening can regulate particle–particle attraction, particle adsorption, and interfacial packing. In oil–water–solid systems, salinity can also change the distribution of surface-active materials between the oil–water interface and solid surfaces. As schematically shown in Fig. 4f, high-salinity conditions can favor adsorption of interfacially active species on the solid surface, whereas low-salinity conditions may enhance their accumulation, partitioning, or microdispersion near the oil–water interface. Although the phase continuity and electrolyte location differ from those in internal-salt-containing W/O emulsions, these observations provide complementary mechanistic evidence for the role of ionic strength in regulating interfacial organization. They further suggest that, in particle- or solid-containing systems, salt effects on interfacial adsorption may involve particle aggregation, solid-surface adsorption, and redistribution of surface-active species in addition to direct ion–interfacial species interactions.

Ion-specific effects further determine how internal salts regulate interfacial adsorption. Monovalent salts, such as NaCl and KCl, mainly act by changing ionic strength and screening electrostatic repulsion near the oil–water interface. In contrast, multivalent salts, especially CaCl_2_ and MgCl_2_, can produce stronger effects because multivalent cations may interact with polar groups in surfactants. Lashkarbolooki *et al.* compared NaCl, CaCl_2_, and MgCl_2_ in acidic crude oil/brine systems and found that the ability to reduce IFT followed the order MgCl_2_ > CaCl_2_ > NaCl.^42^ They attributed the stronger effect of MgCl_2_ to the higher affinity of Mg^2+^ toward oxygen-containing groups in resin molecules, while Ca^2+^ was suggested to complex more readily with larger asphaltene molecules. In another crude oil/brine study, increasing NaCl concentration from 10 to 1000 mM decreased the IFT from 35.9 to 28.7 mN m^−1^, whereas the IFT reduction was even more pronounced for CaCl_2_ brines over the same concentration range.^48^ This stronger effect was attributed to the higher valence of Ca^2+^, which more effectively screens electrostatic repulsion and promotes interactions with polar crude-oil surface-active components than Na^+^.

**Fig. 5:**
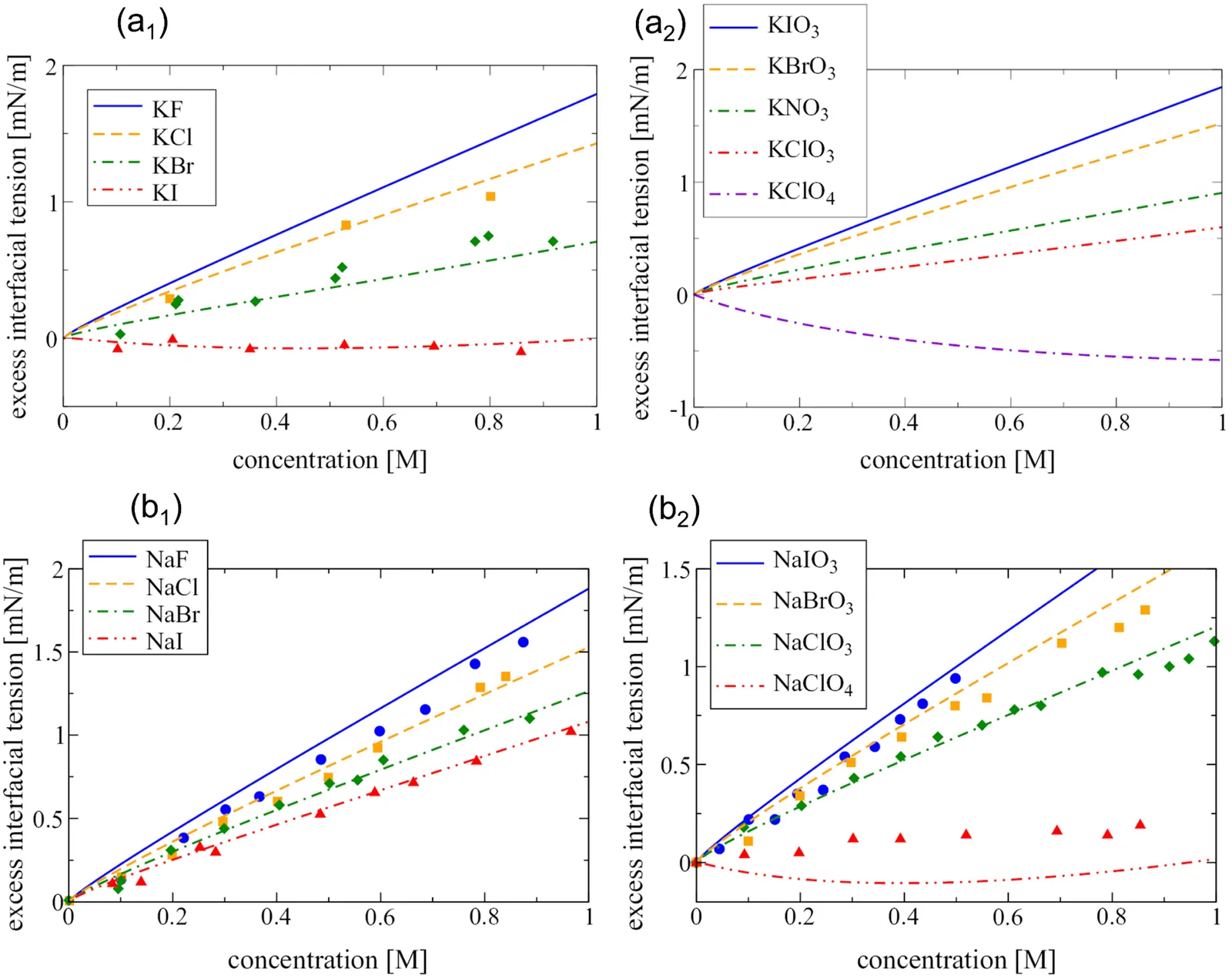
Changes in interfacial tension were induced by different anions in potassium salts (a) and sodium salts (b). Reproduced with permission from ref. 49. Copyright 2014, IOP Publishing.

Anion type can also affect interfacial adsorption and interfacial tension, although its role differs from that of cations. Cations usually interact more directly with polar or negatively charged interfacial groups. In contrast, anions mainly influence interfacial water structure, hydration, polarizability, and ion partitioning near hydrophobic interfaces.^50^ For potassium halides, Santos and Levin found that the interfacial-tension change, relative to the salt-free oil–water interface, decreased in the order KF > KCl > KBr > KI (Fig. 5a).^49^ Across the ionic-strength range of 0–1 M, the IFT change for KI remained very small, reaching only approximately 0.1 mN m^−1^ at 1 M.^49^ Because weakly hydrated ions can adsorb near hydrophobic interfaces and offset the usual salt-induced increase in interfacial tension, this near-zero value indicates stronger interfacial affinity of I^−^. This interpretation was further supported by the ionic density profiles, which showed a significant increase in I^−^ adsorption near the hydrophobic surface. A similar trend was also observed for sodium salts (Fig. 5b). Santos and Levin also compared the effects of IO_3_^−^, BrO_3_^−^, NO_3_^−^, ClO_3_^−^, and ClO_4_^−^.^49^ Their results showed that ClO_4_^−^ behaved distinctly from the other oxyanions. At 1 M, KClO_4_ produced a negative interfacial-tension change, whereas most other oxyanion salts remained positive. The difference between KClO_4_ and the other oxyanion salts was roughly 1–2 mN m^−1^. This contrast was attributed to the strong interfacial adsorption of highly polarizable ClO_4_^−^.

Overall, salinity and ion identity jointly determine salt-regulated interfacial adsorption. At relatively low to moderate ionic strength, typically ranging from 10^−2^ M to several 10^−1^ M, salts can screen electrostatic repulsion among charged or polar interfacial species and promote their adsorption at the oil–water interface, thereby reducing the interfacial tension. When the ionic strength approaches or exceeds ∼1 M, excessive salt may induce interfacial saturation, molecular rearrangement, salting-out, or aggregation, causing an IFT plateau or even an increase. Moreover, salts with stronger electrostatic screening ability or stronger affinity toward polar interfacial groups usually exert more pronounced effects. For cations, divalent ions generally show stronger interfacial regulation than monovalent ions, with reported IFT-reduction ability following MgCl_2_ > CaCl_2_ > NaCl in acidic crude oil/brine systems. For anions, weakly hydrated and highly polarizable ions, such as I^−^ and ClO_4_^−^, show stronger affinity toward hydrophobic interfaces and can partly offset or even reverse the usual salt-induced increase in interfacial tension. These trends indicate that internal-salt effects on interfacial adsorption should be interpreted by considering ionic strength, ion valence, ion-specific affinity, and the composition of interfacially active species together.

## 3 Effects of internal salts on interfacial film strength and viscoelasticity

Internal salts can also regulate W/O emulsion stability by modifying the mechanical properties of the interfacial film.^36^ A stronger interfacial film can better resist droplet coalescence, thereby enhancing emulsion stability.^51^ Interfacial rheology provides more direct information on the resistance of the oil–water interface to compression, expansion, and deformation.^52^ The elastic modulus, viscous modulus, dilatational elasticity, and interfacial compressibility are commonly used to describe the strength and viscoelasticity of the interfacial film. A more elastic or viscoelastic interface usually indicates stronger molecular packing, slower interfacial relaxation, and a higher resistance to interfacial deformation.^53^ Therefore, salt-induced changes in interfacial viscoelasticity are important for understanding how internal salts affect the mechanical robustness of W/O emulsion interfaces.

Salt concentration regulates interfacial film strength and viscoelasticity by changing the adsorption, packing, rearrangement, and aging of interfacially active species at the oil–water interface.^54^ Across the reported concentration windows, increasing ionic strength generally enhances the final mechanical strength of crude oil–brine interfacial films. In Minnelusa crude oil/brine systems, Sarmas-Farfan *et al.* compared NaCl, Na_2_SO_4_, and Na_3_PO_4_ brines over an ionic strength range of 0.006–0.6 M.^55^ Increasing ionic strength produced a moderate increase in interfacial viscoelasticity, generally within approximately 5–25%, depending on the oscillation frequency and aging time. A similar strengthening effect was observed by Alves *et al.* in Brazilian crude oil/brine systems over a higher NaCl concentration range.^56^ When the ionic strength of NaCl brine increased from 0 to approximately 0.86 and then to 4.3 M, the interfacial elasticity increased from 6.06 to 7.71 and 9.65 mN m^−1^, respectively. After 12 h of aging, the inverse compressibility further increased from 9.27 ± 0.13 to 12.43 ± 0.95 and 20.66 ± 2.17 mN m^−1^. These results suggest that salt-induced enhancement of interfacial film strength can extend from low ionic strength to concentrated brine conditions, provided that salt addition promotes interfacial accumulation, molecular packing, and film aging of surface-active components.

**Fig. 6:**
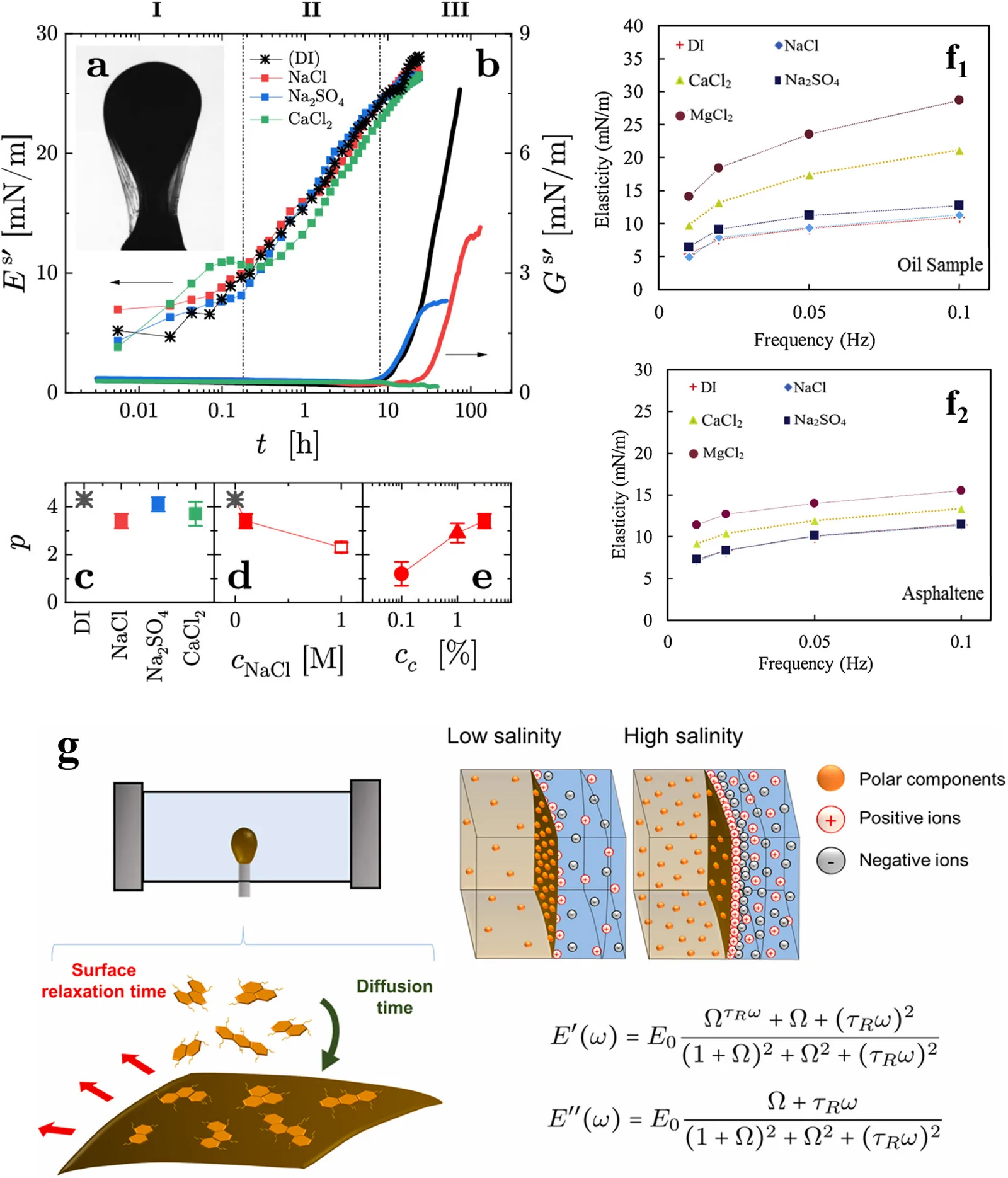
Regulation of interfacial film strength and viscoelasticity by internal salts. (a) Wrinkling of an aged crude-oil droplet after interfacial deformation, indicating the formation of a solid-like interfacial skin. (b) Time-dependent evolution of extensional elastic modulus (*E*^s^′) and shear elastic modulus (*G*^s^′) for crude oil–water interfacial films formed against different aqueous phases. (c–e) Equation-of-state exponent *p* as a function of salt species, NaCl concentration, and crude-oil concentration, respectively, reflecting changes in interfacial softness and molecular organization. Reproduced with permission from ref. 57. Copyright 2022, American Chemical Society. (f) Interfacial elasticity of crude oil and asphaltene systems against 0.1 M NaCl, Na_2_SO_4_, CaCl_2_, and MgCl_2_ brines. Reproduced with permission from ref. 58. Copyright 2019, Elsevier. (g) Transient dilatational-rheology modeling of crude oil–NaCl brine interfaces, showing how salinity regulates the apparent viscoelastic modulus, growth rate, and equilibrium time through adsorption kinetics and surface coverage of polar components. Reproduced with permission from ref. 59. Copyright 2021, Elsevier.

Saad *et al.* demonstrated that ion specificity mainly affects the late-stage network formation of crude oil–water interfacial films.^57^ After 24 h aging, the deflated droplet showed visible wrinkles, indicating the formation of a solid-like interfacial skin (Fig. 6a). Compared with the extensional elastic modulus, *E*^s^′, the shear elastic modulus, *G*^s^′, showed a clearer ion dependence (Fig. 6b). *G*^s^′ remained below the detection limit of ∼0.1 mN m^−1^ during the first two stages, but appeared earlier in DI water and Na_2_SO_4_ systems, was delayed in chloride brines, and was not detected for CaCl_2_ within the experimental time scale. The equation-of-state exponent *p*, which reflects the softness of the interface and its dependence on surface coverage, further varied with salt species (Fig. 6c). Increasing NaCl concentration reduced *p*, indicating altered molecular ordering at the interface, while the dependence of *p* on crude oil concentration confirmed the role of interfacial crowding and lateral interactions (Fig. 6d and e). These results suggest that ion-specific effects are more clearly reflected in late-stage shear elasticity and interfacial softness than in the overall extensional elastic response.

Compared with monovalent salts, multivalent salts generally provide stronger electrostatic screening at the same molar concentration and may further interact with polar groups in resins, asphaltenes, or acidic components. Mahmoudvand *et al.* compared NaCl, Na_2_SO_4_, CaCl_2_, and MgCl_2_ brines and found that divalent cations produced much higher interfacial elasticity than monovalent cations^58^ (Fig. 6f). For crude oil against 0.1 M brines, the interfacial elasticity at 0.1 Hz increased to approximately 20 mN m^−1^ for CaCl_2_ and 28 mN m^−1^ for MgCl_2_, respectively, whereas NaCl and Na_2_SO_4_ gave only about 11–12 mN m^−1^. Similar trends were observed in separated maltene and asphaltene systems, with MgCl_2_ showing the strongest enhancement.

Anion type also affects the development of interfacial viscoelasticity, although the magnitude of this effect is relatively moderate compared with the cation-valence effect discussed above. Sarmas-Farfan *et al.* further isolated the role of anions by fixing the cation as Na^+^ and comparing chloride-, sulfate-, and phosphate-containing brines.^55^ They found that the anion type affected both the final elastic modulus and the buildup rate of the crude oil–brine interfacial film. After 8 h of aging, phosphate-containing brines produced higher elastic moduli, reaching approximately 27–32 mN m^−1^, whereas chloride and sulfate brines generally remained around 24–27 mN m^−1^. The kinetic difference was also evident: the characteristic time for elastic buildup decreased from ∼2.0 h for NaCl to ∼1.4 h for Na_2_SO_4_ and ∼1.1 h for Na_3_PO_4_. These results indicate that the strengthening effect followed the order phosphate > sulfate > chloride, with phosphate-containing brines producing faster-forming and more elastic interfacial films.

Salt-induced changes in interfacial viscoelasticity are closely related to the adsorption, rearrangement, and surface coverage of polar oil components at the oil–brine interface. García and Saraji provided a quantitative description of this process by modeling the transient dilatational rheology of crude oil–NaCl brine interfaces^59^ (Fig. 6g). Their logistic fitting separated the final apparent viscoelastic strength from the buildup kinetics: when NaCl concentration increased from 0.1 to 0.5 wt%, the equilibrium modulus increased from 26.1 to 64.2 mN m^−1^, whereas the growth rate decreased from 0.16 to 0.08 mN m^−1^ h^−1^. This result indicates that salts do not simply accelerate interfacial film formation. Instead, they regulate the adsorption kinetics and limiting surface coverage of polar components, thereby determining the final viscoelastic strength of the interfacial film.

Overall, internal salts regulate interfacial film strength and viscoelasticity by controlling both the final mechanical properties and the time-dependent formation pathway of the interfacial film. Increasing salt concentration can enhance film elasticity and resistance to deformation when it promotes interfacial accumulation, molecular packing, and aging of surface-active components. Ion identity further affects late-stage shear elasticity, interfacial softness, and network formation through ion-specific interactions with polar interfacial species. In the reported crude oil–brine systems, divalent cation salts, especially MgCl_2_ and CaCl_2_, generally strengthen interfacial viscoelasticity more effectively than monovalent salts such as NaCl, while phosphate-containing brines show a stronger film-building effect than sulfate- and chloride-containing brines.

## 4 Effects of internal salts on droplet interactions and coalescence

Droplet coalescence and subsequent phase separation are the final manifestations of W/O emulsion destabilization.^60,61^ The interfacial adsorption and film viscoelasticity discussed above determine the resistance of droplets to coalescence. In addition to these film-mediated effects, internal salts may directly modify droplet interactions through changes in interfacial charge and electrostatic interactions.^62,63^ Therefore, this section focuses on how interfacial charge affects droplet interactions, thereby providing further insight into the salt-regulated stability of W/O emulsions.

Electrostatic droplet interactions are more commonly considered in O/W emulsions, where ions are present in the continuous aqueous phase and classical electrostatic screening concepts can be directly applied. In W/O emulsions, however, water droplets are separated by a low-dielectric oil phase with generally low ionic solubility, making electrostatic interactions less straightforward. Nevertheless, significant electrostatic repulsion can also arise between water droplets in oil. Zwanikken *et al.* theoretically showed that unequal ionic solvation energies between water and oil can induce charge separation at the interface and generate charged water droplets.^64^ For micron-sized droplets, the predicted charge can reach approximately 10–1000 elementary charges, with appreciable electrostatic interactions occurring in oils with dielectric constants of approximately 4–10.

**Fig. 7:**
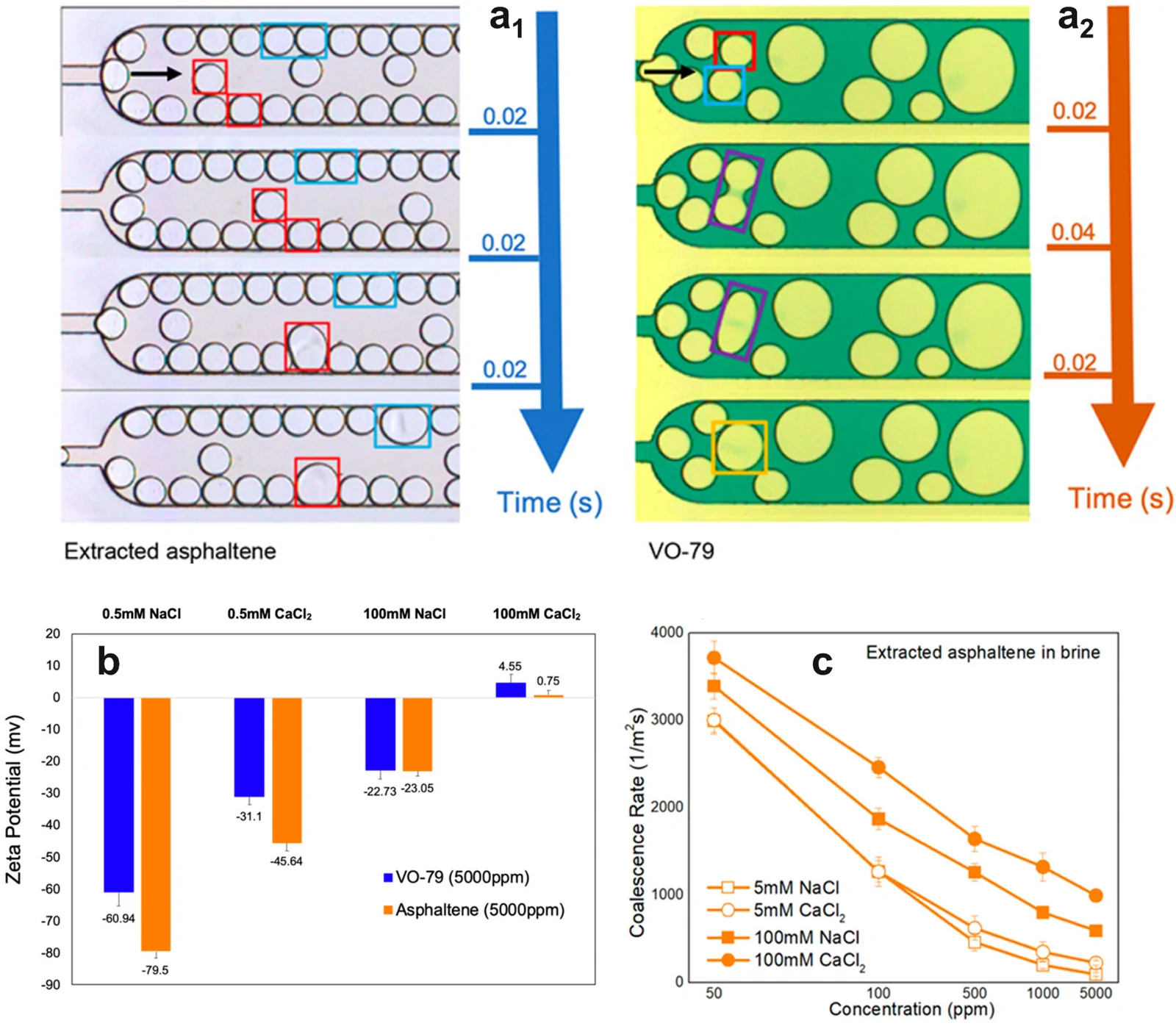
Effect of internal salts on interfacial charge-mediated droplet interactions and coalescence in W/O emulsions. (a) Microfluidic visualization of brine-droplet coalescence in oil. (b) Zeta potential of asphaltene- and VO-79-stabilized oil/brine interfaces under different ionic-strength conditions. (c) Coalescence rates of W/O emulsions under different brine conditions. Reproduced with permission from ref. 65. Copyright 2020, American Chemical Society.

Zhang *et al.* directly visualized the coalescence of brine droplets in oil and quantified the coalescence rate to evaluate W/O emulsion stability^65^ (Fig. 7a). They found that increasing salt concentration from the millimolar level to 100 mM generally accelerated droplet coalescence, with the coalescence rate increasing by approximately 50–100% in selected systems (Fig. 7c). Moreover, CaCl_2_ brines resulted in higher coalescence rates than NaCl brines, suggesting that divalent Ca^2+^ more effectively screens or neutralizes the negative charge at oil–water interfaces than monovalent Na^+^. Zeta-potential measurements further showed that asphaltene-stabilized interfaces were more negatively charged at low ionic strength (Fig. 7b). At high ionic strength, the charge difference was reduced through electrostatic screening; in 100 mM CaCl_2_, the zeta potential even became slightly positive. These changes indicate that salts modify the interfacial electrostatic state and may thereby weaken electrostatic repulsion between approaching droplets.

Taken together, internal salts regulate brine droplet coalescence by modifying the electrostatic state of the oil–water interface and the resulting interactions between approaching droplets. Increased ionic strength or specific ions may weaken electrostatic repulsion, facilitating closer droplet approach and subsequent coalescence.^66^ This electrostatic pathway acts together with the interfacial adsorption and film-mediated effects discussed above, making droplet coalescence an integrated outcome of multiple salt-regulated processes.

## 5 Bulk-property effects of internal salts on W/O emulsion stability

In addition to interfacial mechanisms, internal salts can regulate W/O emulsion stability through bulk-property effects. Salt concentration strongly affects the physical state of the dispersed phase and the final emulsion. For example, brine density will affects gravitational sedimentation and droplet accumulation. Internal salts may also modify emulsion viscosity, thereby affecting droplet migration, collision frequency, film drainage, and coalescence kinetics. Meanwhile, salt solubility defines the formulation window of high-density or mixed brines, because crystallization or precipitation may disrupt emulsion homogeneity. Therefore, this section discusses the roles of density, emulsion viscosity, and salt solubility in salt-regulated W/O emulsion stability.

Increasing internal salinity increases the density of dispersed brine droplets and thus the density difference between the aqueous and oil phases. According to Stokes' law, this increased density difference favors faster gravitational sedimentation of isolated droplets, although deviations may occur in concentrated or non-Newtonian emulsions. A higher brine density therefore tends to accelerate the downward migration and accumulation of water droplets in W/O emulsions.^67^ Accelerated sedimentation can increase the local droplet concentration and contact probability, thereby favoring subsequent coalescence and water separation. This effect becomes more pronounced in high-density brine systems. For example, saturated NaCl solution has a density of approximately 1.20 g mL^−1^ at 25 °C, while commercial CaBr_2_ brine with a density of about 1.70 g mL^−1^ is commonly used as a high-density brine. Mixed ZnBr_2_/CaBr_2_ brines used in completion fluids can further reach 1.74–2.16 g mL^−1^. Nour and Yunus reported that increasing NaCl concentration promoted water separation in water-in-crude-oil emulsions. After 1800 min, the separated water increased from 57% without salt to 68% at 1.5% NaCl and 91% at 5.5% NaCl.^68^ However, the density effect should not be interpreted as the only stability mechanism. Ling *et al.* found that oilfield W/O emulsion stability increased with salinity over five days, and this trend was independent of whether NaCl or CaCl_2_ was used.^69^ This indicates that density-driven sedimentation may be counterbalanced by other factors.

The apparent viscosity of W/O emulsions is another bulk-property factor that affects kinetic stability. Higher emulsion viscosity can slow droplet migration and delay phase separation, but it also increases flow resistance and processing difficulty. Unlike brine density, emulsion viscosity is not determined by salinity alone. It is jointly controlled by water content, droplet size distribution, interfacial structure, droplet–droplet interactions, and the viscosity of the continuous oil phase. After emulsification, dispersed water droplets increase hydrodynamic resistance and often make the system more viscous than the original oil.^70,71^ The role of ion type was further demonstrated by studies comparing NaCl, CaCl_2_, Na_2_SO_4_, MgCl_2_, and MgSO_4_.^72^ When the salt concentration increased from 1 × 10^3^ to 1 × 10^4^ ppm, the apparent viscosity increased for all systems, but the extent varied markedly with salt type. MgCl_2_ caused the strongest increase, from approximately 1950 to 2450 cP, followed by CaCl_2_ from 1300 to 1560 cP, whereas NaCl showed the weakest effect, increasing only from about 750 to 1120 cP. Na_2_SO_4_ and MgSO_4_ gave intermediate viscosities. When the salt concentration was further increased to around 5 × 10^4^ ppm, the viscosity decreased to varying degrees, indicating a nonmonotonic dependence on salt concentration. These results suggest that ion identity, rather than salinity alone, can significantly modify emulsion viscosity, probably by altering droplet–droplet interactions, interfacial structure, and the strength of the dispersed droplet network.

**Fig. 8:**
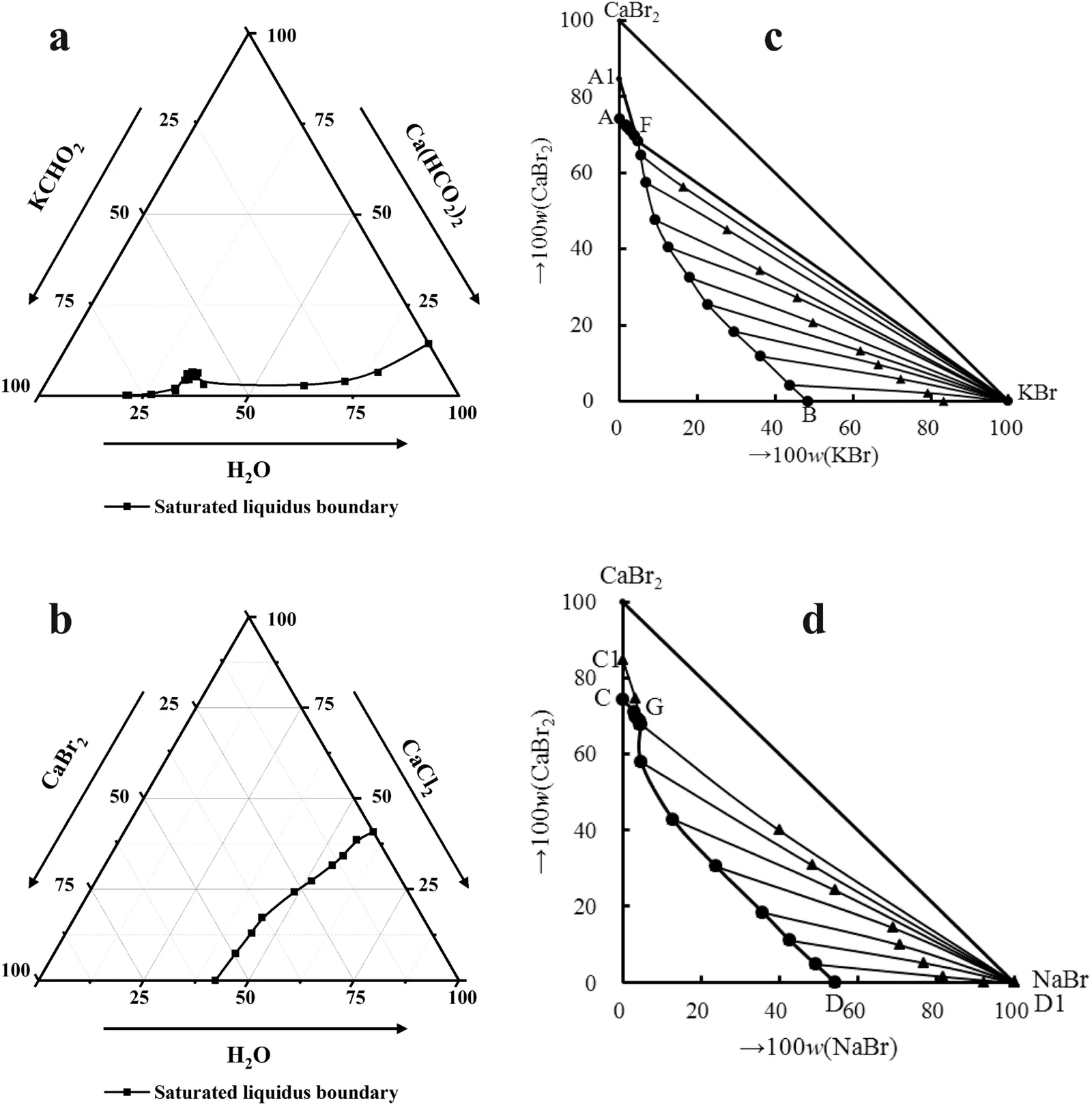
Solid–liquid equilibrium constraints of representative mixed brine systems. (a) Ternary solubility diagram of the KCHO_2_–Ca(CHO_2_)_2_–H_2_O system at 25 °C, redrawn using data from the IUPAC-NIST Solubility Database, NIST Standard Reference Database 106.^73^ (b) Solid–liquid phase equilibrium of the CaCl_2_–CaBr_2_–H_2_O system at 288.15 K, redrawn from ref. 74. Copyright 2017, American Chemical Society. (c and d) Solid–liquid equilibrium diagrams of the KBr–CaBr_2_–H_2_O and NaBr–CaBr_2_–H_2_O systems at 348 K. Reproduced with permission from ref. 75. Copyright 2015, American Chemical Society.

At high salt concentrations, crystallization or precipitation can alter the composition and physical state of the internal brine phase, thereby compromising the homogeneity of W/O emulsions. Salt solubility and phase equilibria therefore define important formulation constraints for high-salinity systems. As shown in Fig. 8a, the KCHO_2_–Ca(CHO_2_)_2_–H_2_O system contains different liquidus branches at 25 °C.^73^ The saturated liquid may equilibrate with Ca(CHO_2_)_2_, KCHO_2_, or the double salt K_4_Ca(CHO_2_)_6_, depending on composition. This indicates that mixed organic brines can precipitate not only simple salts but also double salts when the formulation approaches the solubility boundary. A similar constraint exists for inorganic halide brines. Fig. 8b shows the CaCl_2_–CaBr_2_–H_2_O system, where the same Ca^2+^ cation is combined with different anions.^74^ The liquidus boundary illustrates that replacing Cl^−^ with Br^−^ changes the accessible high-salt composition range. Fig. 8c and d further show the KBr–CaBr_2_–H_2_O and NaBr–CaBr_2_–H_2_O systems.^75^ These diagrams contain composition-dependent crystallization fields, demonstrating that the addition of monovalent bromide salts can shift CaBr_2_-rich brines into different precipitation regions. For example, in the KBr–CaBr_2_–H_2_O system, the equilibrium solution at invariant point F contained 68.34 wt% CaBr_2_ and 4.70 wt% KBr, indicating a CaBr_2_-rich crystallization boundary. Therefore, high-density brines should not be treated as infinitely soluble internal phases. Their formulation window is governed by salt composition, temperature, and solid–liquid equilibria.

In practical high-density completion fluids, crystallization temperature is also a critical design parameter. Liimatta *et al.* noted that many deepwater reservoirs require brines with densities above 1.76 g cm^−3^, while conventional divalent brines may suffer from pressure-induced crystallization.^76^ Their zinc-free brine with a density of approximately 1.80 g cm^−3^ had a true crystallization temperature below −20.6 °C, whereas the last crystal to dissolve temperature of a CaBr_2_ solution at the same density was close to ambient temperature. For W/O emulsions, crystallization or precipitation would alter the composition and physical state of the internal phase and disrupt emulsion homogeneity. Such changes are undesirable for maintaining a uniform emulsion structure and should therefore be avoided in practical formulations. However, their specific effects on emulsion stability remain insufficiently investigated and may be system-dependent.

## 6 Challenges and perspectives

Based on the mechanisms and representative findings discussed in Sections 2–5, this section further synthesizes the differences and unresolved issues among existing studies and identifies the major challenges in understanding internal-salt-regulated W/O emulsion stability.

The fundamental challenge is the coupled and competing nature of salt effects, which manifests as strong system dependence, apparently contradictory conclusions, and limited comparability among studies.^50,54^ Internal salts can simultaneously regulate interfacial adsorption, interfacial film mechanics, electrostatic interactions, and bulk properties,^69,77^ with these pathways sometimes acting in opposite directions (Table 1). For example, salt-induced interfacial adsorption and film strengthening may enhance resistance to coalescence, whereas charge screening can weaken electrostatic repulsion and promote droplet coalescence. The relative contribution of these pathways depends strongly on salt concentration, ion identity and valence, oil composition, emulsifier type, water content, and temperature. Consequently, variations in system composition and experimental conditions can shift the relative importance of individual mechanisms, leading to different or even opposite stability responses. This system dependence also limits direct comparison among studies and makes it difficult to isolate individual salt effects or establish generally applicable conclusions.

**Table 1:** Competing stabilizing and destabilizing effects of internal salts on W/O emulsion stability and their likely dominant conditions

Salt-regulated pathway	Stabilizing effect	Destabilizing effect	Conditions favoring either effect
Interfacial adsorption	Enhanced adsorption and interfacial coverage; multivalent ions generally exert stronger effects	Reduced effective interfacial coverage	Moderate salinity (ionic strength from 10^−2^ M to several 10^−1^ M) favors adsorption; excessive salinity (>1 M) may induce saturation, or aggregation
Interfacial film mechanics	Stronger packing and higher film elasticity	—/system-dependent	Governed by ion identity, emulsifier/oil composition, salinity, and aging
Electrostatic droplet interactions	Stronger electrostatic repulsion	Charge screening and enhanced coalescence; multivalent ions generally produce stronger screening	Low ionic strength favors repulsion; high ionic strength and multivalent ions favor screening
Brine density	—/limited direct stabilizing contribution	Faster gravitational droplet migration	More pronounced at large oil–brine density differences
Emulsion viscosity	Slower droplet migration and collision	Lower viscosity promotes droplet mobility	Governed by salt composition, water content, and emulsion structure
Solubility/crystallization	Homogeneous internal phase	Crystallization and disrupted homogeneity	High salt loading and proximity to crystallization boundaries favor precipitation

The second major challenge arises from the technical limitations of current characterization methods. On the one hand, many key microscopic and interfacial processes remain difficult to observe directly, so current mechanistic interpretations often rely on indirect experimental evidence and inference.^62^ On the other hand, realistic conditions, including high temperature and pressure, strong shear, and optically opaque petroleum systems, are difficult to reproduce within conventional characterization platforms. As a result, simplified oils, emulsifiers, brines, or transparent model systems are often employed. These limitations restrict both the direct verification of proposed mechanisms and their validation under practically relevant conditions.

**Fig. 9:**
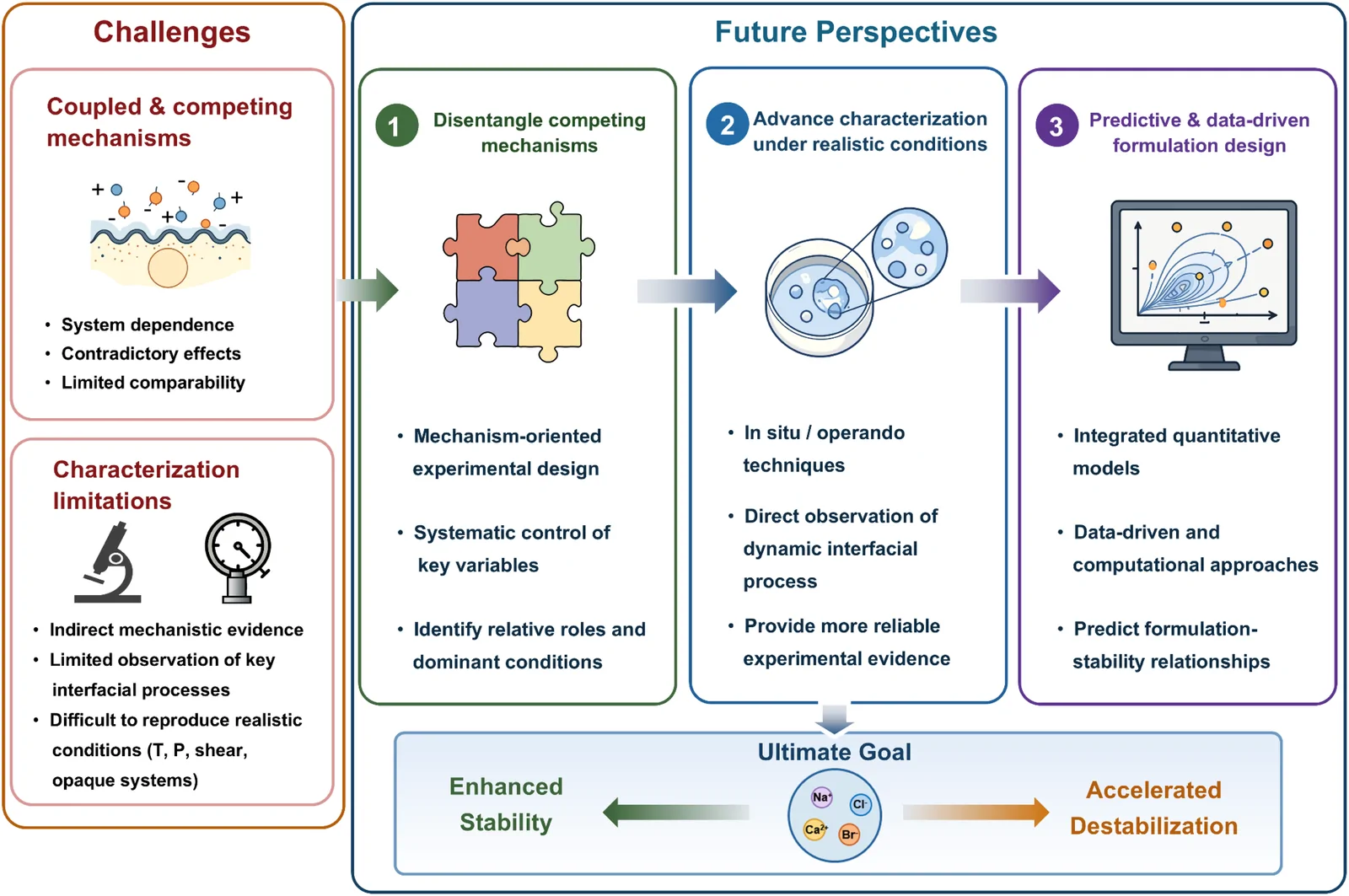
Challenges and future perspectives for internal-salt-regulated W/O emulsions.

To address these challenges, future research should move toward an integrated and predictive framework that connects brine composition, competing mechanisms, operating conditions, and macroscopic emulsion stability. As summarized in Fig. 9, three directions are particularly important: disentangling competing mechanisms, advancing characterization under realistic conditions, and developing predictive formulation strategies.

First, future studies should focus on disentangling the relative roles of competing salt-regulated mechanisms and identifying the conditions under which individual mechanisms dominate. This requires mechanism-oriented experimental designs with systematic control of key variables to distinguish different regulatory pathways. Such efforts would provide a clearer mechanistic understanding of salt-regulated W/O emulsion stability.

Second, advanced experimental and characterization techniques should be developed to directly probe salt-regulated processes under realistic conditions. For example, advanced *in situ* and *operando* techniques could enable direct observation of dynamic interfacial processes under practical conditions, providing more reliable experimental evidence for understanding salt-regulated emulsion stability.

Finally, future research should move toward predictive and intelligent formulation design based on a clearer understanding of competing salt-regulated mechanisms. Advanced computational and data-driven approaches could be used to establish relationships between brine composition, physicochemical conditions, and emulsion stability, enabling more precise formulation optimization. Such strategies could ultimately support the targeted regulation of W/O emulsions toward either enhanced stability or accelerated destabilization.

## 7 Conclusions

This review summarizes the regulation of W/O emulsion stability by internal salts from a mechanism-oriented perspective. Salt effects are considered across the stabilization process, from interfacial film formation and resistance to droplet coalescence to macroscopic emulsion stability, together with bulk-property effects. Internal salts regulate these processes through ion-mediated interfacial interactions and changes in bulk-phase properties, thereby affecting interfacial adsorption, film mechanics, droplet interactions, and droplet migration. Importantly, these regulatory pathways are coupled and can exert competing effects on emulsion stability. The overall stability response therefore depends on the relative contributions of these pathways under specific physicochemical conditions, which explains the strong system dependence of salt effects.

Current understanding, however, remains limited by the difficulty in distinguishing the relative contributions of competing mechanisms and by the lack of direct observation of microscopic processes under realistic conditions. Future research should combine molecular simulation, advanced interfacial characterization, realistic-condition testing, and phase-equilibrium-based formulation design. Such efforts will help build a more predictive framework for the rational control of salt-containing W/O emulsions in separation processes, petroleum production, and functional emulsion formulations.

## Author contributions

Panhong Yu: conceptualization, investigation, data curation, formal analysis, writing – original draft. Yang Xue: formal analysis, writing – review & editing. Chao Li: formal analysis, visualization, writing – review & editing. Sunan Wang: investigation, data curation. Huaike Li: methodology, formal analysis, writing – review & editing. Tie Geng: visualization, writing – review & editing. Hong Sui: supervision, funding acquisition, project administration, writing – review & editing. Lin He: supervision, conceptualization, writing – review & editing, funding acquisition, project administration.

## Conflicts of interest

There are no conflicts to declare.

## Data Availability

No primary research results, software or code have been included and no new data were generated or analysed as part of this review.
